# 

**Published:** 1888-09-15

**Authors:** 


### To Residents, Secretaries, and Matrons.

THE Editor will be glad to have the Regulations and each Report as issued by Asylums, Hospitals, Convalescent and Nursing Institutions, as well as those of other Charities, and an early copy in duplicate of all Appeals and Festival Notices, etc., addressed to *The Editor, The Lodge, Porchester Square, London, W.* Any superintendent, secretary, matron, or other chief official who regularly sends such information may be registered, on application to the Publisher, to receive free of cost a cover for binding THE HOSPITAL in half-yearly volumes.

### Notice to Advertisers and Subscribers.

All Advertisements and Business Communications should be addressed to The Publisher, *not to the Editor*, THE HOSPITAL, 38, Old Jewry, E.C.
A Scale of Charges will be found in the Advertisement Sheets, page iii.

Examination justifies the conclusion that, as regards the recoveries in asylums which have been established during any considerable period—say twenty years—a proportion of much less than 40 per cent. of the admissions is, under ordinary circumstances, to be regarded as a low proportion, and one much exceeding 45 per cent. as a high proportion.

### THE CHILDREN'S CORNER.

PRIZES.
FOR MOST CORRECT ANSWERS TO PUZZLES.
This Commenced October 1st, 1887.
One Pound a Month.
A PRIZE OF THREE GUINEAS
to the Competitor who shall have sent in the largest number of correct or best answers during the twelve months.

### Third Week Puzzles.

No. 9. Double Acrostic.—1. An Alpine Summit. 2. Napoleon Buonaparte's birth-place. 3. An extensive district of North America. 4. A river of South Wales. 5. An ancient province of France. Initials and finals form the names of two islands.

No. 10. Square Word.

```
. . . .    1. A rock.
. . . .    2. To value.
. . . .    3. A particle.
. . . .    4. Precious stones.
```

No. 11. Enigma.—How many sons have you? said one gentlemen to another. He answered, "Six, and each son has a sister." How many children had he?

No. 12. Geographical Charade.—My *first* and *second* together form a familiar name; my *third* is a part of the human body; and my *whole* a town in Scotland.

### Results of First Week Puzzles.

No. 1.—Geographical Enigma.—Morocco.
No. 2.—Charade.—Fare—well=Farewell.
No. 3.—Diamond Puzzle.

```
            P
        D   I   N
    H   O   N   E   Y
V   I   N   E   G   A   R
P   I   N   E   A   P   P   L   E
    C   R   O   P   P   E   D
        C   A   P   E   R
            A   L   E
                E
```

No. 4.—Biographical Charade.—Play—fair=Playfair.

| Marks. | Sept. 1st. | Total First Week. |
|---|---|---|
| A. C. K. | 4 | 4 |
| Gem | 4 | 4 |
| G. M. M. | 3 | 3 |
| Scrooge | 3 | 3 |
| Thanet | 4 | 4 |
| Tynesider | 3 | 3 |
| Judy | 3 | 3 |
| Primrose | 3 | 3 |
| J. W. M. | 3 | 3 |
| Agamemnon | 4 | 4 |
| G. E. R. | 3 | 3 |
| Happy Eliza | 3 | 3 |
| Penelope | 3 | 3 |
| Ikye Mo | 1 | 1 |
| Boadicea | 3 | 3 |

### Conditions.

I. Every paper sent in must be clearly written, and one side only must be used. All competitors must mark at the head of their papers the number of their competition.

II. Each paper must be signed by the author with his or her real name and address. A *nom de plume* may be added, if the writer does not desire to be referred to by us by his real name. In the case of all prizewinners, however, the real name and address will be published.

III. All communications relating to prize competitions should be addressed to "The Prize Editor, The Hospital, 38, Old Jewry, London, E.C." Competitors are requested not to include in letters, etc., to the Prize Editor communications on matters of other business. All letters must be fully prepaid.

IV. The Prize Editor of The Hospital is the sole judge of the competitions, and his decision is in all cases final and without appeal.

